# Effect of açai berry extract application on the bond strength to the bleached enamel using an experimental etch-and-rinse adhesive

**DOI:** 10.4317/jced.59758

**Published:** 2022-12-01

**Authors:** Lamiaa M. Moharam, Haidy N. Salem, Shahinaz N. Hassan

**Affiliations:** 1 Restorative and Dental Materials Department, National Research Centre, Giza, Egypt

## Abstract

**Background:**

To evaluate the bleached enamel shear bond strength (SBS) after antioxidants application using an experimental adhesive.

**Material and Methods:**

Sixty sound human molars had their roots removed then cut into buccal and lingual halves and mounted in acrylic blocks. Enamel bleaching was done using an in-office chemical bleaching agent. Specimens were arbitrarily allocated into 12 groups (n=10) regarding the three experimental levels of the study: two antioxidant agents [10% açai berry extract, 10% sodium ascorbate prepared gels (applied for 15-min) and the control group (no antioxidant application)], two adhesive materials [commercial etch-and-rinse (ER) adhesive and an experimental ER adhesive] and two post-bleaching SBS testing times [24-h and 2-w]. Specimens were restored with a nano-hybrid resin composite restoration then stored in distilled water at 37◦C till SBS testing.

**Results:**

Three-way ANOVA showed that the antioxidant agents, adhesive materials and the post-bleaching SBS testing times had a statistically significant effect on the bleached enamel SBS. Açai berry extract groups recorded the highest mean values, while the control groups demonstrated the least values with a statistically significant difference between the antioxidant agents’ groups. Commercial ER adhesive recorded statistically significant higher SBS values than the experimental adhesive and the 2-w post-bleaching SBS testing time demonstrated statistically significant higher SBS values than the 24-h groups.

**Conclusions:**

Açai berry extract is a powerful antioxidant agent, that has the potential to instantly restore the bleached enamel depleted SBS. The commercial adhesive has successfully restored the depleted SBS of the bleached enamel than the tested experimental adhesive.

** Key words:**Antioxidant application, açai berry extract, etch-and-rinse adhesives, experimental adhesive, shear bond strength.

## Introduction

A remarkable increase in patients’ awareness of esthetic dentistry has notably evolved in the past decade as many patients of different ages are keen to pursue for a brighter smile. Over the years, several strategies have been promoted for the management of stained teeth, which requires an esthetic intervention. Nowadays, teeth bleaching presents a simple, nonviolent, and conservative approach for the treatment of teeth staining. “Chromogens” are the organic pigments accountable for teeth staining and they consist of coupled double bonds (1). The market is flooded with different bleaching agents that consist of hydrogen peroxide as the active ingredient in the bleaching procedure. It has low molecular weight and high infiltration capacity through the interprismatic spaces of the enamel as well as the dentinal tubules. Hydrogen peroxide interacts with the “chromogens” molecules through an oxidization reaction, and subsequently colorless complexes are finally created (2). Nevertheless, the bleaching process is known to decrease the bond strength of enamel and dentin. Reactive oxygen and free radicals are commonly produced as byproducts of the bleaching procedure, that remain on the tooth surface. Such byproducts offset the created free radicals from the resin monomers’ polymerization reaction and interfere with the resin monomer diffusion during the bonding process (3). Additionally, an increased adhesive failure incidence and altered resin tags formation as well as variant Ca/P ratio have been reported in bleached teeth (4). This will eventually result in inferior bond strengths of the promptly restored bleached teeth, thus negatively affecting the restoration durability (5). Clinically, it is recommended to wait for 1-3 w before performing any restorative treatment, so that the residual oxygen will decay by time, and the enamel can be remineralized by saliva. To avoid such waiting periods as well as some of the side effects of the bleaching procedure, several antioxidant materials have been proposed. The gold standard of these antioxidants is “sodium ascorbate”. It is considered the most famous and most examined antioxidant of non-natural origin in the literature (6). Still, it suffers from some drawbacks, as it has a short shelf life, and it is a highly unsTable compound that rapidly oxidizes (7). Accordingly, the application of various natural antioxidant agents, such as grape seed extract, green tea, and aloe vera have been proposed as an alternative to the required pre-restoration waiting time. They were reported to give a favorable outcome as reversers of such adverse effects triggered during the bleaching procedure. These natural extracts contain diverse phenolic compounds, which govern their free radical scavenging potential (8). It was found that antioxidants can successfully neutralize the reactive oxygen and the free radicals of the bleaching procedure, thus restoring the compromised enamel bond strength immediately (9).

A worldwide interest in “açai berry” (Euterpe oleracea) has been largely growing due to its beneficial properties. A significant attention has focused on its high antioxidant potential, that has been accredited for its compounds of polyphenols. Additionally, its anthocyanins content was found to have a photo-protective influence by direct elimination of the reactive oxygen during the photo-oxidative stress. Açai berry was found to contain increased amounts of proanthocyanidins (PAs) among other non-anthocyanin polyphenol and phenolic acids, that were accountable for its significant antioxidant property (10).

As the frequency of performing immediate post-bleaching adhesive procedures has increased lately (6), the need for a potent adhesive material to ensure a successful and durable bond with the bleached enamel, became essential to overcome the variable complications of teeth bleaching. Thus, an immediate and uncompromised enamel and dentin bonding would be established effectively, upon antioxidants application prior to the adhesive restorations. Hereafter, the objective of this study is to investigate the effect of application of prepared antioxidant agent gels (10% açai berry extract, and 10% sodium ascorbate) on the SBS of the bleached enamel using an experimental ER adhesive at two SBS testing times (24-h and 2-w). The tested null hypotheses were: (a) The application of 10% açai berry extract and 10% sodium ascorbate gels will not affect the SBS to the bleached enamel. (b) The prepared experimental ER adhesive will have no influence on the bonding performance of the bleached enamel. (c) Post-bleaching waiting periods of 24-h and 2-w will not affect the bonding performance of the bleached enamel following the application of the prepared antioxidant gels.

## Material and Methods

-Selected Materials

Two experimentally prepared antioxidant agents; [10% açai berry extract and sodium ascorbate gels], two etch and rinse adhesives; [an experimental etch and rinse (ER) adhesive and a commercial ER adhesive (Solobond M)], a chemical bleaching agent [WHITEsmile POWER WHITINING] and a nanohybrid resin composite [Filtek Z350 XT] were used in the study. The materials brand name, description, composition and their manufacturers are listed in Table 1.

**Table 1:**
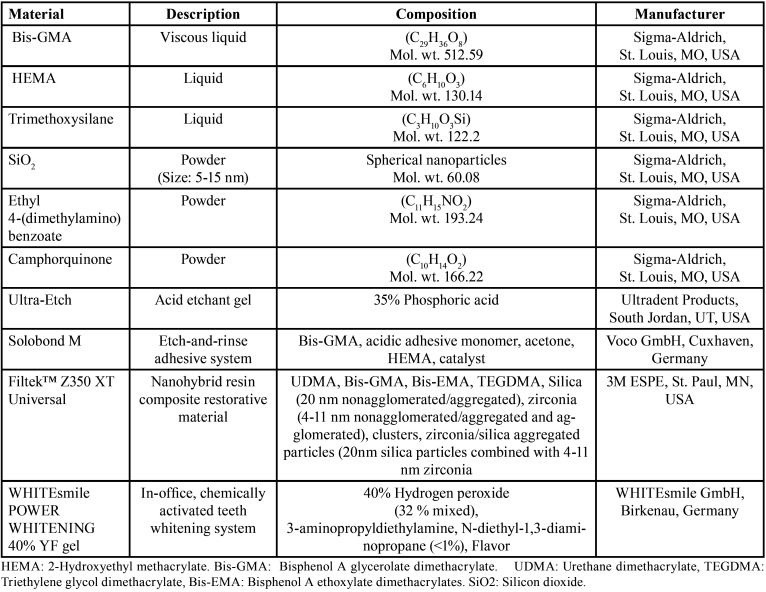
The materials used in the study and their composition, description, and manufacturer.

-Experimental design and groups allocation

**Figure 1:**
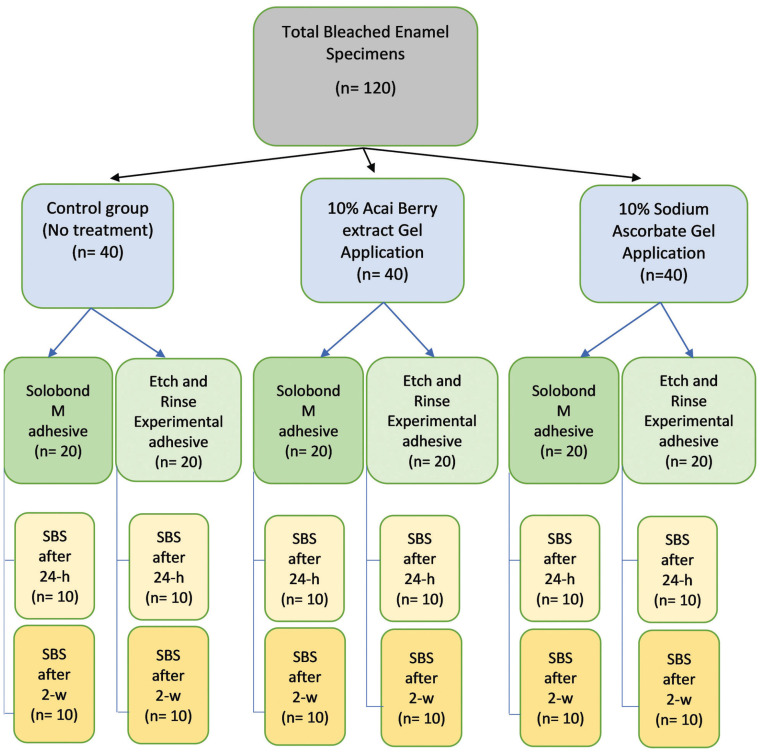
Flowchart of the specimens grouping of the current study.

Sixty sound extracted human molars were selected, and each one was cut into buccal and lingual halves. The 120 cut specimens were bleached and randomly allocated into three main groups (40 specimens each), representing the two prepared antioxidant agent gels [10% açai berry extract gel, 10% sodium ascorbate gel] and the control group (no treatment). Each main group was further divided into two subgroups (20 specimens each) according to the applied ER adhesive materials [Solobond M (commercial ER adhesive) and the experimental ER adhesive]. Then each subgroup was divided into two divisions (n=10) representing the two SBS testing times [24-h and 2-w]. A flowchart for the specimens grouping is demonstrated in Figure 1.

Sample size calculation was performed using R statistical package, version 2.15.2 (26-10-2012). Copyright (C) 2012 - The R Foundation for Statistical Computing. One-way ANOVA analysis showed that a total sample size of 10 samples was accepted to detect a mean difference between the study groups with a power of 80% and a two-sided significance level of 5%.

Preparation of the experimental adhesive

Bis-GMA monomer of 70 wt% concentration was mixed with 30 wt% HEMA monomers, then a binary photo-polymerization system composed of 0.5% ethyl 4-dimethylamine benzoate and 0.5% camphorquinon was added to the previous mixture to make it photo-curable (11). Afterwards, 10 wt% acetone and 10 wt% ethanol solvents (PioChem, Giza, Egypt) were added to the experimental adhesive. The mixture was stirred for 2-h uninterruptedly in a magnetic stirrer (Wisestir MSH-300, Witeg Labortechnik, Wertheim, Germany) in presence of a small piece of magnet for proper homogenization of the mixture and to guarantee complete dissolution of the monomers into the added solvents. Alternatively, acetic acid (PioChem, Giza, Egypt) was steadily added to a 40 ml of 70% ethanol solution in a few drops to adjust its pH to 3˗4, then a silane coupling agent of 3 wt% concentration was added to the previously pH-adjusted solution, then the solution was continuously stirred for 1-h to finally obtain a silane coupling agent of an increased hydrolysis rate. SiO2 nanoparticles were added to the mixture, then it was centrifuged for 30-min. The excess ethanol solvent was removed, and the precipitate was dried at 105°C in an air-pressure oven (12). Thereafter, the 0.1 wt% silanized SiO2 nanoparticles was added to the prepared experimental adhesive, that was stirred for 2-h until all nanoparticles were completely dispersed. The prepared adhesive was kept in a light-proof container at 4◦C till usage.

-Preparation of antioxidant agent gels

According to Kimyai and Valizadeh (13), an amount of 0.25 mg of açai berry extract powder (Wild Harvested Açai Berry Powder, Nature Restore Inc Products, Santon, CA, USA) and another 0.25 mg of sodium ascorbate (Sigma-Aldrich, St. Louis, MO, USA) were separately dissolved in 2.5 ml of purified water at room temperature, to obtain 10%-concentration solutions of açai berry extract and sodium ascorbate respectively. The two solutions were further stirred in the magnetic stirrer for 5-min to ensure complete dissolution of each powder into the purified water. Then two Carbopol® gels (2.5% wt/wt) comprising of 10% açai berry extract and 10% sodium ascorbate were prepared by dissolving the Carbopol® resin powder (Carbomer 940, Sanarelab LTD, KH, London, GB) with moderate mixing in the purified water containing açai berry extract and sodium ascorbate respectively. Each mixture was stirred using the magnetic stirrer till thickening is evident. Next, the thickened mixtures were neutralized by adding thriethanolamine (PioChem, Giza, Egypt) drops until transparent gels were obtained. The thriethanolamine amount was adjusted to obtain gels of a pH=7.

-Selection and preparation of the teeth

Sixty sound human molars were extracted and cleansed from any residual tissues and debris then stored at 4◦C in 0.1% thymol solution till use. The roots of the teeth were cut 2-mm beyond the enamel-cementum junction using low-speed, two side-cutting diamond discs. Each molar tooth was cut mesio-distally, to separate the lingual and buccal surfaces. The two halves of each tooth were rinsed ultrasonically in distilled water for 10 min, and then dried with gentle air jets. For the ease of manipulation, the teeth halves were partially embedded in chemical cured acrylic resin blocks (Acrostone, Acrostone Dental Factory, Egypt), with the enamel facing upwards. Silicon carbide abrasive papers of 1000-1200 grits were used to achieve homogenous and even enamel surfaces (3).

-Bleaching procedure

As per the manufacturer’s commands, the in-office chemical activated WHITEsmile bleaching agent was applied in a 1.5-2 mm thick layer on the enamel surfaces of the prepared specimens and it was left uninterrupted for 15-min. Then the bleaching gel was removed from the enamel surfaces with cotton rolls. That process was repeated three times for a total of 45-min bleaching procedure. Upon completion of the bleaching procedure, the specimens were thoroughly rinsed with water for 30-s (14).

-Application of the antioxidant agent gels

The prepared 10% açai berry extract and sodium ascorbate gels were applied to the bleached enamel surfaces of the assigned specimens using micro brushes in a 3-mm thickness gel layer and left undisturbed for 15-min (14). Then the gels were removed using cotton rolls, and the specimens were thoroughly rinsed under running water for 30-s (6).

-Application of the restorative systems

Ultra-Etch™ etchant gel was applied to the enamel surfaces of the prepared specimens for 15-s according to the manufacturers’ directions. Next, the gel was rinsed with copious amounts of water for 30-s to remove any remnants of the etchant gel. The two ER adhesives (Solobond M and the experimental adhesive) were applied to the bleached enamel surfaces of the assigned groups, according to the manufacturer’s recommendation of the commercial adhesive (Solobond M), using micro brushes in two to three coats for 30-s followed by gentle air dryness for 5-s, to modify the consistency and the film thickness of the adhesive layer. The adhesive systems were then photo-cured using LED light curing unit (Elipar S 10, 3M ESPE, USA) for 20-s. The output intensity of the curing unit (≥ 1000/cm2) was checked sporadically using a handheld radiometer (Demetron 100, Kerr Corporation, Orange, CA, USA). Transparent plastic rings (2-mm height x 1.8-mm diameter) were placed at the maximum convexity of the bleached specimens, and Z 350 Universal resin composite material was inserted inside the plastic rings in one increment. Celluloid strips were placed on the top of the restorations. According to the manufacturer instructions, the resin composite was photo-cured for 20-s (3). A sharp blade was used to cut the plastic rings and remove any flashes extending beyond the base of the composite discs. The specimens were kept in tight-seal containers in distilled water at 37◦C until the SBS measurement was accomplished at the specified testing times, either after 24-h or after 2-w (15).

-Shear bond strength test (SBS)

Specimens were attached to the lower jig of the universal testing machine (Instron®, Model 3345, Instron Instruments, Buckinghamshire, UK) and a chisel bladed metallic attachment at the upper jig of the machine was positioned as close as possible to the restoration/enamel interface and the test was run at 0.5 mm/min cross head speed till failure. The maximum force was calculated in MPa. SBS was calculated by dividing the peak load at failure by the specimen’s surface area using the machine computer software (BlueHill® Universal, Instron Testing Software, Buckinghamshire, UK).

-Statistical Analysis

The mean and standard deviation values were calculated for each group in each test. Data were explored for normality using Kolmogorov-Smirnov and Shapiro-Wilk tests, and the investigated data showed a parametric (normal) distribution. One-way ANOVA followed by Tukey post-hoc test was used to compare between more than two groups in non-related samples. The Independent sample t-test was used to compare between two groups in non-related samples. On the other hand, Paired sample t-test was used to compare between two groups in related samples. Three-way ANOVA tests was used to test the interactions between different variables. The significance level was set at *P* ≤ 0.05. Statistical analysis was performed with IBM® SPSS® Statistics Version 20 for Windows.

## Results

**Figure 2:**
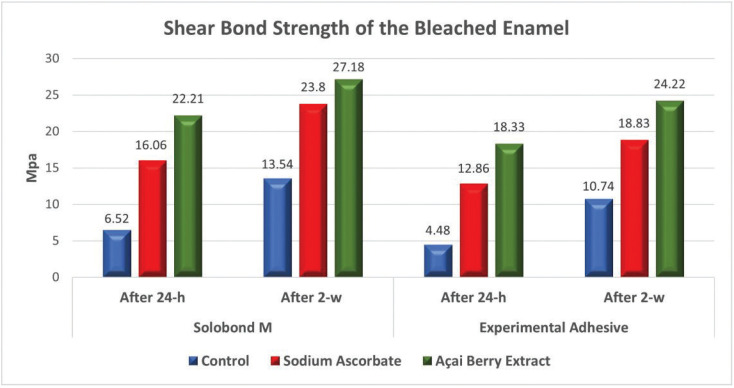
Bar chart showing the effect of the investigated antioxidant agents on the SBS of the bleached enamel at the two SBS testing times for both tested adhesive materials.

The three main variables of the study [the two antioxidant gels (10% açai berry extract and 10% Sodium ascorbate gels); the two adhesive materials (Solobond M and the experimental ER adhesives); and the two post-bleaching SBS testing times (24-h and 2-w)] were investigated for their interaction, and it was shown that the two tested antioxidant agents, the two adhesive materials and the two SBS post-bleaching testing times had a statistically significant effect on the SBS of the bleached enamel. Figure 2 showed the three-way ANOVA analysis for the effect of the investigated antioxidant agents [10% açai berry extract gel, 10% sodium ascorbate gel] on the SBS of the bleached enamel at 24-h and 2-w SBS post-bleaching testing times, for both tested adhesive materials [Solobond M and the experimental adhesive]. A statistically significant difference between the control group (no antioxidant treatment), the 10% sodium ascorbate and 10% açai berry extract groups was revealed (*p*<0.001), with the highest mean value recorded for the 10% açai berry extract groups, while the least mean value was found in the control groups. For the effect of the tested adhesive materials on the SBS of the bleached enamel at both SBS post-bleaching testing times within each antioxidant agent; Table 2 showed a statistically significant difference between the adhesive materials [Solobond M and experimental adhesive] at *p*<0.001. The highest mean value was recorded for Solobond M groups, while the least mean value was recorded for the experimental adhesive groups. For the effect of the two SBS post-bleaching testing times (24-h and 2-w) on the SBS of the bleached enamel for both tested adhesives within each applied antioxidant agent; Table 3 showed a statistically significant difference between the 24-h groups and 2-w groups at *p*<0.001. The highest mean value was recorded for the 2-w groups, while the least mean value was found in 24-h groups. The results obtained demonstrated that the highest SBS mean value was recorded for the group of Solobond M adhesive treated with the 10% açai berry extract gel at 2-w post-bleaching SBS testing time (27.18±0.62), while the least SBS mean value was recorded for the control group with the ER experimental adhesive at 24-h post-bleaching SBS testing time (4.48±0.38) at *p*<0.001.

**Table 2:**
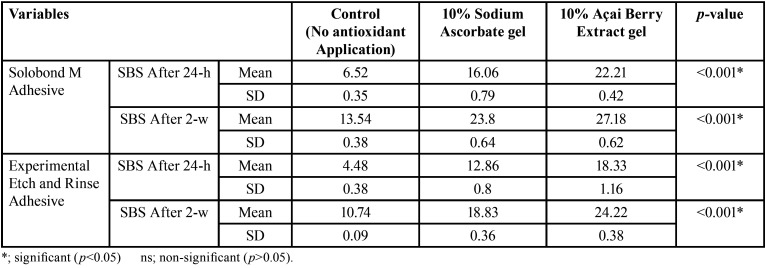
The effect of the different adhesive materials on the shear bond strength (SBS) of the bleached enamel at the two SBS testing times within each antioxidant agent gel.

**Table 3:**
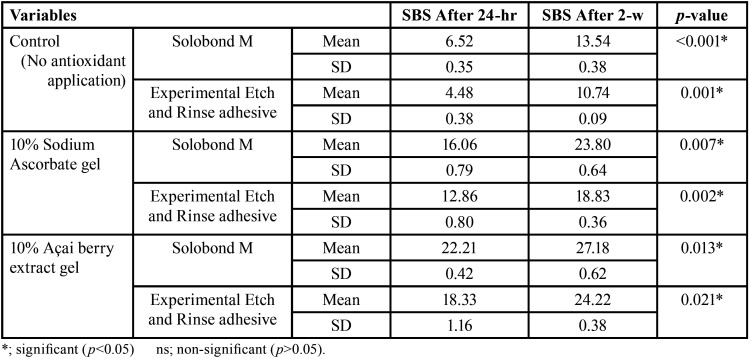
The effect of the shear bond strength (SBS) of the bleached enamel at the two SBS testing times for the tested antioxidant agents within each adhesive material.

## Discussion

Nowadays, merging the dental bleaching procedures with modern adhesive restoratives to accomplish the desirable esthetic outcomes has turned out to be a regular practice. As frequently, it became necessary to restore the decayed teeth with direct resin composite restorations, immediately after the completion of the bleaching procedure. Moreover, the suggested 1- 3 w post-bleaching waiting period for restoration placement, is not often feasible or achievable in most clinical circumstances (15,16).

Consequently, the immediate post-bleaching restoration placement would compromise the bond strength of the bleached enamel. Therefore, the urge to reinstate the depleted bond strength of the freshly bleached enamel has increased significantly. In this context, numerous natural antioxidant agents have been investigated for their potential effect upon restoring the remarkably decreased bond strength of the newly bleached enamel. The results of the current study revealed a significant positive consequence of the examined antioxidant agents on the depleted SBS of the bleached enamel at both tested time intervals (24-h and 2-w) for both investigated adhesives. Thus, the proposed null hypotheses were rejected.

Antioxidants application on the bleached enamel prior to resin composite restorations, was found to significantly offset the deleterious effect of the reactive oxygen and free radicles that are produced as byproducts during the bleaching procedure (17). Natural antioxidants such as açai berry and grape seed extract contains high levels of polyphenols and oligomeric PAs complexes, both complexes are responsible for their substantial antioxidant potential (18). This could be owed to the compositional hydroxyl groups of the PAs and polyphenol compounds, which interact with the hydrogen ions that directly bind to the free radicals produced by the bleaching process, and finally neutralizing them (19).

On the other hand, sodium ascorbate was found to allow for the free-radical polymerization of resin composite restorations, and thus counteracting the negative effects of the bleaching-produced residual oxygen (20). Furthermore, the findings of the present study showed that açai berry could restore the depleted SBS of the bleached enamel more efficiently than the sodium ascorbate, which could be related to their significantly elevated natural antioxidant capacity. These findings agreed with Olmedo *et al.* (21) and Gogia *et al*., (22) who concluded that the natural oligomeric PA Compounds have a prodigious free radical scavenging ability. Nevertheless, their antioxidant potential has been demonstrated to be 50 times more powerful than “vitamin C”, from which sodium ascorbate is derived.

According to the results of the current study, the resin composite restorations directly placed following teeth bleaching (after 24-h) showed lower SBS values compared to those placed after 2-w. These findings were in accordance with other studies (20,23,24), they concluded that the adhesive enamel performance had a direct relation with the bleaching process, as the generated reactive oxygen of the bleaching procedure could actively contest with the free radicals produced from the polymerization process of the subsequently applied adhesive restorative system. Moreover, a significant reduction in the degree of resin monomers conversion was evident directly following teeth bleaching, which could be directly accountable for the depleted bond strength of the bleached enamel as a result of weak hybrid layers creation (25,26). Likewise, a diminished resin infiltration into the bleached enamel surface was pronounced, that could be owed to the surface changes produced by the oxygen remnants, increased voids formation at the enamel/adhesive interface and within the hydroxyapatite crystals as well as impaired Ca/P ratio (27).

The findings of the present study showed that, a waiting period of 2-w before placement of the resin composite restorations, had a positive effect on the SBS to the bleached enamel. This could be explained by the tendency of the reactive oxygen produced during the bleaching procedure, to decay and decline in amount by time (28). Moreover, subjecting the bleached teeth *in vivo* to the saliva of the oral cavity can be an adjunct that aids to restore the previously depleted bond strength, as the saliva has other antioxidant agents and could induce enamel remineralization that is vital for increasing its mechanical properties (23). Furthermore, antioxidant agents were proven to induce a probable post-bleaching enamel remineralization, as remineralization was found to be directly related to the antioxidants neutralization capacity of the reactive oxygen generated during bleaching process (20,25).

Novel interesting strategies with multiple possible applications have been proposed to overcome the ordeal of teeth bleaching side effects. These could be represented in using dental adhesives and resin composites that contain natural antioxidant agents as a part of their components or by using these antioxidants in conjunction with the contemporary and new adhesive systems to reverse the adverse effects of bleaching on the bond strength to bleached teeth. Moreover, the need for more efficient dental adhesives capable of increasing the overall bond strength of the enamel and dentin is a great deal. Therefore, an ER experimental adhesive was investigated in the current study for its potential effect on the SBS of the bleached enamel in conjunction with natural antioxidant agent application. Yet, the results of the current study showed that both antioxidant agents had presented significantly higher SBS values at both post-bleaching testing times (24-h and 2-w) for the commercial ER adhesive (Solobond M), than the experimental ER adhesive prepared for the study. On the other hand, Abraham *et al*. (29) compared the effect of PAs-containing natural antioxidant agent application on the bond strength of the bleached enamel using ER adhesive system and one-bottle self-etch (SE) adhesive system. They concluded that PAs application can raise the bleached enamel bond strength for the ER adhesive systems. They owed their results to the pronounced etching pattern of the ER adhesive and its adequate pH. In this context, the results of the experimental adhesive used in the current study was quite promising, as the experimental adhesive was able to reinstate the depleted SBS of the bleached enamel, but not to the level of the commercial adhesive. Nevertheless, combining the antioxidants application with the experimental adhesive yielded a significantly increased SBS values. On the other hand, these finding disagreed with those of Gelmini *et al*. (7), they investigated the effect of an experimental SE adhesive incorporated with grape seed extract on the SBS of bleached enamel and their results showed no statistically significant difference between the control group (adhesive without antioxidant) and grape seed extract-incorporated adhesive group while the least SBS values were recorded for the sodium ascorbate-incorporated adhesive group. Such inconsistencies between the results of the studies could be related to the difference in the adhesive systems as well as the bleaching agent used in both studies. An ER adhesive system along with 40% hydrogen peroxide bleaching agent was used in the present study while they used SE adhesive with 45% carbamide peroxide bleaching agent. Additionally, they owed the decreased SBS of the bleached enamel to the carbamide peroxide bleaching agent that might have slowly produced less frequent free radicals than hydrogen peroxide during the bleaching procedure, which might have facilitated the neutralization of the bleaching free radicles with the adhesive incorporated antioxidants in such low contact time between the antioxidants and the bleached enamel before light curing of the adhesives’ layers.

Consequently, one can possibly validate antioxidants application as an actual worthwhile substitute to the required waiting time for the immediate restorations placement and rehabilitation procedures that had to occur after the bleaching procedures, regarding their probability to counteract the momentary adverse effects following teeth bleaching. Moreover, natural antioxidants are quite able to fully restore the altered redox potential that occurred due to the produced reactive oxygen as well as free radicals generated on the bleached enamel surface. Hence, they are quite capable of restoring the immediate bond strength of the bleached enamel. Further research is essential to inspect the impact of different types of antioxidant agents, their concentration, and application time on the jeopardized bleached enamel bond strength.

## Conclusions

Based upon the limitations of the present *in vitro* study it could be concluded that; the prepared 10% açai berry extract gel was a powerful antioxidant agent that had the potential to instantly restore the depleted bond strength of the bleached enamel, thus eliminating the proposed waiting time required for restorations placement. Moreover, both prepared antioxidant agent gels were able to reverse the adverse effect of bleaching on the immediate SBS with the two tested adhesives. The commercial ER adhesive had a significantly higher effect on the SBS of the bleached enamel compared to the experimental ER adhesive.
